# Role of complete blood cell count parameters in the diagnosis of neonatal sepsis

**DOI:** 10.1186/s12887-022-03471-3

**Published:** 2022-07-13

**Authors:** Minichil Worku, Melak Aynalem, Sirak Biset, Berhanu Woldu, Tiruneh Adane, Abiye Tigabu

**Affiliations:** 1 Department of Medical Microbiology, School of Biomedical and Laboratory Sciences, College of Medicine and Health Sciences, University of Gondar, Gondar, Ethiopia; 2 Department of Hematology and Immunohematology, School of Biomedical and Laboratory Sciences, College of Medicine and Health Science, University of Gondar, 196 Gondar, Ethiopia

**Keywords:** Hematological parameters, Diagnostic tool, Neonatal sepsis, Gondar, Ethiopia

## Abstract

**Background:**

Neonatal sepsis is one of the leading causes of neonatal morbidity and mortality in developing countries like Ethiopia. The investigation of neonatal sepsis needs the application of inclusive diagnostic tools. Therefore, this study aimed to assess the role of CBC parameters in diagnosing neonatal sepsis.

**Methods:**

A comparative cross-sectional study was conducted from September 2020 to November 2021 at the University of Gondar Comprehensive Specialized Hospital. A total of 250 neonates were included using a convenient sampling technique. A structured questionnaire and a data collection sheet were used to obtain the socio-demographic and clinical characteristics of the study participants. A venous blood sample was collected for CBC and blood culture tests. Epi-Info Version 7 and SPSS Version 25 were used for data entry and analysis, respectively. The data distribution was checked by the Shapiro-Wilk test. Then, an independent t-test was conducted to compare CBC parameters, and the significant parameters were recruited for the ROC curves analysis. The Younden index test was used to determine the cutoff point for the sensitivity and specificity. A *p*-value of <0.05 was considered statistically significant.

**Results:**

Out of 250 study participants, 144 (57.6%) were males, with a median age of 6 days (IQR = 4 days). Early-onset and late-onset sepsis were developed in about 29.6% (37/250) and 70.4% (88/250) of the neonates, respectively. The TLC and ANC parameters were significantly lower in cases than in control groups. The TLC, Hgb, lymphocyte count, and ANC parameters have a sensitivity of 64.8, 68, 33.6, and 49.6%, respectively. Their specificity in the diagnosis of neonatal sepsis was 64.8, 53.6, 83.2, and 90.4%, respectively.

**Conclusion:**

Total leucocyte count, ANC, and platelet count all showed significant associations with neonatal sepsis. Besides, the TLC, ANC, and platelet counts had good sensitivity and specificity in diagnosing neonatal sepsis. Therefore, these parameters can be used as a diagnostic tool for neonatal sepsis in resource-limited areas.

## Background

Neonatal sepsis is a public health problem globally. It is the leading cause of morbidity and mortality in the neonatal age, especially in middle and lower-income countries like Ethiopia [1, 2]. According to a world health organization report, nearly 1.6 million (20%) of newborn deaths in Africa and Asia are caused by sepsis [3]. However, accurate diagnosis for the clinician is a difficult task and needs the application of inclusive diagnostic tools [4].

Neonatal sepsis is an infection involving the blood stream within 4 weeks of life. It can be categorized as early-onset sepsis (EOS) if it occurs within 72 hours of delivery and late-onset sepsis (LOS) if it occurs after 72 hours of birth [5, 6]. The common pathologic agents of EOS are Group B *Streptococcus*, *Escherichia coli (E. coli)*, coagulase-negative *Staphylococcus* (CoNS), *Haemophilus influenza* (H. *influenza)*, and *Listeria monocytogenes (L. monocytogenes)*. These agents are transmitted from the maternal genitourinary system to the neonate during delivery [7, 8]. Furthermore, the most common LOS causative agents are CoNS, *S. aureus*, and *Candida*, transmitted through environmental contamination during invasive medical care procedures that damage the mucosal membrane [4, 7].

Diagnosis of neonatal sepsis commonly uses patient history, physical examination, signs and symptoms, and laboratory tests [4]. However, diagnosing neonatal sepsis is not a simple activity. Neonates with bacteremia may be asymptomatic or exhibit symptoms similar to other diseases [7]. Thus, clinical laboratory tests play a crucial role in diagnosing neonatal sepsis. Currently, among neonatal sepsis diagnostic tools, blood culture is considered the gold-standard method. However, in blood culture testing, isolation of causative pathogens is not always possible and needs a longer turnaround time (TAT). Besides, other advanced techniques can be used to diagnose neonatal sepsis. But these advanced methods are costly and require an advanced laboratory set up [5, 7]. Instead of blood culture and advanced laboratory tests, a complete blood cell count (CBC) with differential count can be used to diagnose neonatal sepsis. These tests are technically simple, cheap in cost, have a shorter TAT, and do not require advanced laboratory infrastructure and well-trained laboratory personnel [9].

For the diagnosis of neonatal sepsis, total leucocyte count (TLC), absolute neutrophil count (ANC), immature neutrophil to total neutrophil ratio, and platelet count are commonly used [10]. Besides, neutropenia has better specificity than neutrophilia and leukocytosis [11]. However, the neutrophil count can be affected by other infections, maternal hypertension, gestational age, mode of delivery, and altitude of location of birth [12]. Studies showed that leucopenia, neutropenia, and thrombocytopenia counts were significantly associated with LOS [13, 14].

However, there is limited information about hematological parameters as a diagnostic tool for neonatal sepsis, especially in developing countries like Ethiopia. Therefore, the main aim of this study was to determine the role of hematological parameters as a diagnostic tool for the identification of neonatal sepsis among suspected neonates. In addition, we tried to see changes in hematological parameters in cases and control groups, and we also assessed antimicrobial sensitivity patterns for the identified bacteria.

## Materials and methods

### Study setting and study population

A hospital-based comparative cross-sectional study was conducted from September 2020 to November 2021 to assess the role of hematological parameters as a diagnostic tool for the identification of neonatal sepsis among suspected study participants. This study was conducted at the University of Gondar Comprehensive Specialized Hospital (UoG-CSH), which is located in Gondar town. According to the 2007 central statistics agency of Ethiopia, there was a total population of 207,044 living in the town [15]. The projected population number in 2020 is estimated at 362,000 [16]. The UoG-CSH is a teaching hospital and it is the oldest academic institution in Ethiopia that provides medical services for more than 7 million people in Gondar and neighboring regions. The hospital has different departments, including pediatrics, internal medicine, surgery, gynecology, and laboratory [17].

A total of 125 culture-positive cases and 125 culture-negative controls, aged from 1 to 28 days old, were included. Study participants with neonatal sepsis and attending the neonatal intensive care unit treatment center were considered as a source population. Whereas, study participants who had confirmed sepsis by blood culture were considered part of the study population. The inclusion criteria for this study was that a study participant who had suspected neonatal sepsis be included in this study. Neonates with preterm birth, a pregnancy terminated using drugs or surgical intervention after implantation and before the embryo or fetus has become independently viable, congenital anomalies, an inborn metabolic error, severe jaundice and respiratory distress syndrome, surfactant deficiency, extremely low birth weight newborns, mothers with pregnancy-induced hypertension, and neonates with asphyxia were excluded from the study.

### Operational definitions

Neonatal sepsis: is an infection involving the bloodstream in neonates less than 28 days old.

Septicemia suspected: is neonates having fever, not taking feed, respiratory distress, low birth weight, convulsion, poor cry, or meconium-stained liquor [2].

EOS: septicemia within 3 days of delivery [5].

LOS: septicemia within 3–28 days of delivery [5].

### Data collection and laboratory procedures

A structured questionnaire and a data collection sheet were used to obtain socio-demographic, clinical characteristics, and laboratory data. The data was collected by professional nurses and laboratory technologists. First, the data collectors have been trained by principal investigators about the aim of the study. Then, the data collection procedure was collected under the supervision of principal investigators.

### Socio-demographic and clinical data collection

A pre-tested structured questionnaire was used to collect sociodemographic data. To maintain the consistency of the data collection tool, the questionnaire was first written in the English language, then converted into the local language (Amharic), and finally back into the English language. To improve the quality of the data, the questionnaire was pre-tested in a poly-health center before the actual data collection.

### Blood sample collection for CBC and blood culture

A total of 2 mL of venous blood samples were collected by the vacutainer blood collection technique. About 1 ml venous blood sample was dispensed into a blood culture test tube for the blood culture test. Besides, the rest of 1 mL of the blood was dispensed into a test tube that contained Ethylene Diamine Tetra Acetic acid anticoagulant, which is used to count the CBC. To maintain the quality of blood samples, they were collected by trained professional laboratory technologists by following all standard blood collection procedures.

### CBC analysis

The CBC analysis was performed using a Sysmex KX-21 hematology analyzer. Sysmex KX-21 is a multi-parameter blood cell counter. To count blood cells, the auto-machine uses an impedance principle. The impedance principle uses a constant electric current that is passed through a blood sample and reagent solution to determine the changes in electrical resistance that occur when blood cells pass through the detection aperture [18]. To keep the quality of the CBC analyzer to commercially prepared known blood samples (normal, low, and high), background checks and machine maintenance were done as per the manufacturers’ instructions and as per the clinical laboratory institute for standardization standard [19].

### Blood culture analysis

An experienced laboratory technician collected a blood sample aseptically from each study participant who developed signs and symptoms of sepsis at the time of diagnosis. The collected blood samples were then transferred into sterile tryptic soy broth (Oxoid Ltd., Basingstoke, UK) and incubated at 37 °C. Daily, indicators of bacterial growth such as turbidity, hemolysis, or clot formation were observed for seven days. Tryptic soy broths with signs of bacterial growth were gram-stained and sub-cultured on blood agar, chocolate agar, MacConkey agar, and mannitol salt agar (Oxoid Ltd.). The inoculated plates were then aerobically incubated for 18–24 hours at 37 °C and the isolates obtained were identified using standard microbiological methods. Gram-negative bacteria (GNB) were identified using a series of biochemical tests such as indole production, urease production, decarboxylase production, oxidase production, triple sugar iron agar (sugar fermentation), H_2_S production, citrate utilization, and motility tests. On the other hand, Gram-positive bacteria (GPB) were identified based on catalase production, coagulase production, hemolytic pattern, optochin test, bacitracin test, bile esculin test, and salt tolerance test. To ensure the sterility of the culture media, nearly 5% of the prepared culture media were chosen at random and incubated aerobically for 24 hours at 37 °C to maintain the quality of the blood culture results. In addition, known strains of *S. aureus* (ATCC 25923) and *E. coli* (ATCC 25922) were inoculated to check the performance of the prepared culture media. Inoculation of culture media and colony characterization were checked by an experienced microbiologist. The microbial sensitivity test was performed by inoculating 5% of sheep blood supplemented with fastidious bacteria across the whole surface of Muller Hinton agar (Oxoid, UK) with a bacterial suspension having a turbidity of 0.5 McFarland turbidity standard. Finally, using a modified Kirby-Bauer disk diffusion method, the antibiotic susceptibility profile of isolates was evaluated, and the findings were interpreted using the clinical laboratory standards institute recommendation [20, 21].

### Data entry and analysis

First, the data was entered and cleared by Epi-Info Version 7 and then exported into the statistical package for social science (SPSS) Version 25 software for analysis. Data distribution was checked by the Shapiro-Wilk test. Continuous numeric variables were expressed by the mean and standard deviation for normally distributed data and the median and interquartile range (IQR) for skewed data. Categorical variables were described using frequency and percentages. Furthermore, to compare hematological parameters, an independent t-test was conducted, and significant parameters were considered for the receiver operating characteristic (ROC) curve analysis, which was used to determine the sensitivity and specificity of each parameter. Finally, the Youden index test was used to determine the cutoff value for the sensitivity and specificity.

### Ethics approval

Ethical clearance was obtained from the ethical review committee of the school of biomedical and laboratory sciences, University of Gondar. Written informed consent from the parents of neonates was obtained. Additionally, the confidentiality of information was assured, and this study was conducted following the declaration of Helsinki.

## Results

### Sociodemographic and clinical characteristics of study participants

**Fig. 1:**
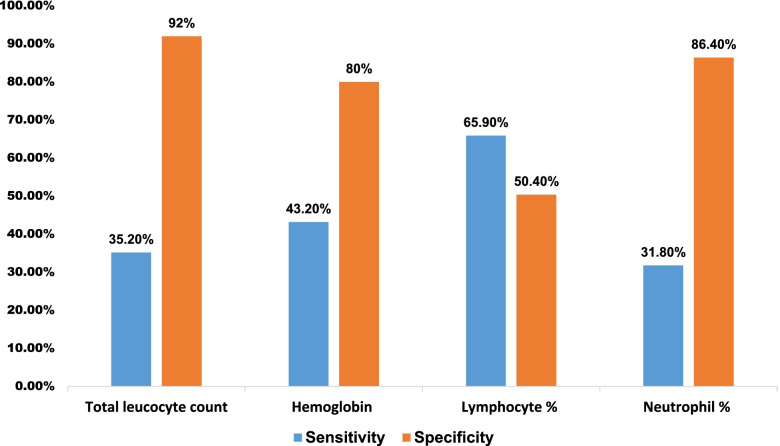
Sensitivity and specificity of hematological parameters for the diagnosis of neonatal sepsis

In this study, a total of 250 study participants were included. Of the total culture positive cases, about 57.6% (144/125) were males, and the median age of the study participants was six days (IQR = 4 days), with an age range of 1 to 28 days. The majority of the study participants, 37.2% (93/250), were within the age range of 7 to 28 days. Figure 1

The EOS and LOS were developed in 29.6% (37/250) and 70.4% (88/250) of the total study participants, respectively. Approximately 94% (235/250), 17.2% (43/250), 9.2% (23/250), and 6.4% (16/250) had an axial fever of more than 38 degrees Celsius, an increased respiratory rate, were suspected of pneumonia, and suspected of meningitis, respectively (Table 1).

**Table 1:** Sociodemographic and clinical characteristics of study participants at UoG-CSH Northwest Ethiopia in 2021, *N* = 250

Variables	Categories	Case	Control
Sex	Female	43	63
	Male	82	62
Age in days	< 3	37	51
	4–7	37	32
	7–28	51	42
Types of sepsis (*n* = 125)	EOS	37	0
	LOS	88	0
Axial fever (>38oc)	Yes	4	11
	No	121	114
Hypothermia	Yes	2	4
	No	123	121
Increased respiratory rate	Yes	31	12
	No	94	113
Suspicious of pneumonia	Yes	11	12
	No	114	113
Suspicious of meningitis	Yes	3	13
	No	122	112
Antibiotic therapy	Yes	68	77
	No	40	56
Type of organism isolated	*S. aureus*	10 (4.0%)	0
	*P. mirabilis*	3 (1.2%)	0
	*S. viridans*	8 (3.2%)	0
	*Providencia* species	2 (0.8%)	0
	*Citrobacter*	1 (0.4%)	0
	*Shigella* species	2 (0.8%)	0
	*K. pneumoniae*	26 (10.4%)	0
	*E. coli*	7 (2.8%)	0
	CoNS	49 (19.6%)	0
	*Enterococcus* species	6 (2.4%)	0
	*Acinetobacter* Species	3 (1.2%)	0
	*K. ozanae*	2 (0.8%)	0
	*E. cloacae*	2(0.8%)	0
	NLF-GNR	4 (1.6%)	0

*EOS* early onset symptom, *LOS* late onset symptom, *UTI* urinary tract infection, *CoNS* coagulase negative staphylococci, *NLF-GNR* non lactose fermenter gram negative rods

### Laboratory findings of study participants

In the current study, CoNS 19.6% (49/125) was the most common isolated bacteria, followed by *K. pneumoniae* 10.4% (26/125), *S. aureus* 4.0% (10/125), and *S. viridians* 3.2% (8/125). On the other hand, the proportion of GPB was 58.4% (73/125) and CoNS 67.1% (49/73) was the predominant type. The proportion of the GNB was 41.6% (52/125) and *K. pneumoniae* 50% (26/52) was the dominant isolate (Table 1).

The overall antibiotic resistance rate of bacterial isolates causing neonatal sepsis was 65.2% and ranged from 29.3 to 88.9%. Cephalosporin (86.05%), co-trimoxazole (83.7%), fluoroquinolones (78.6%), penicillin (74.4%), and aminoglycosides (59.3%) had high levels of resistance, while meropenem (26.3%), vancomycin (25%), and chloramphenicol (17.4%) had low levels of resistance (Table 2).

**Table 2:** Antimicrobial susceptibility pattern of bacterial isolates causing neonatal sepsis at the UoG-CSH

Isolates		Antibiotic																	Total (%)
		PEN	AMX	AUG	PIP	OXA	CAZ	CRO	CEF	CXT	CIP	MEM	COT	GEN	TOB	AMK	CAF	VAN	
*S. aureus*	S	2	–	–	–	3	–	–	–	3	–	–	1	3	1	0	3	–	16 (44.4)
	R	8	–	–	–	5	–	–	–	2	–	–	0	3	0	2	0	–	20 (55.6)
*P. mirabilis*	S	–	0	1	–	–	1	1	–	–	–	0	1	3	–	0	2	–	9 (52.9)
	R	–	2	0	–	–	0	0	–	–	–	2	2	0	–	2	0	–	8 (47.1)
*S. viridians*	S	4	0	–	–	–	–	4	1	0	–	–	–	–	–	–	1	7	17 (60.7)
	R	3	2	–	–	–	–	2	1	2	–	–	–	–	–	–	0	1	11 (39.3)
*Providencia* spp.	S	–	0	–	0	–	0	0	–	–	–	2	2	2	–	–	–	–	6 (42.9)
	R	–	2	–	2	–	2	2	–	–	–	0	0	0	–	–	–	–	8 (51.1)
*Citrobacter*	S	–	–	1	–	–	–	–	–	–	1	1	0	0	–	1	–	–	4 (66.7)
	R	–	–	0	–	–	–	–	–	–	0	0	1	1	–	0	–	–	2 (33.3)
*K. pneumoniae*	S	–	4	0	2	–	0	0	0	–	4	15	0	9	5	3	4	2	48 (29.3)
	R	–	1	17	4	–	11	14	7	–	10	7	23	15	2	1	4	0	116 (70.7)
*E. coli*	S	–	0	–	–	–	0	0	0	–	0	3	3	0	–	1	2	–	9 (20)
	R	–	3	–	–	–	3	1	7	–	6	0	3	7	–	6	0	–	36 (80)
*Enterococcus* spp.	S	3	–	–	–	–	–	–	–	–	–	–	–	–	–	–	6	3	12 (70.6)
	R	2	–	–	–	–	–	–	–	–	–	–	–	–	–	–	0	3	5 (29.4)
*Acinetobacter* spp.	S	–	–	0	–	–	0	0	0	–	0	3	0	1	1	1	1	–	7 (41.2)
	R	–	–	1	–	–	1	1	2	–	1	0	2	2	0	0	0	–	10 (58.8)
*K. ozaenae*	S	–	–	0	0	–	0	0	0	–	0	1	–	0	–	1	–	–	2 (14.3)
	R	–	–	2	2	–	2	1	1	–	1	1	–	1	–	1	–	–	12 (85.7)
*E. cloacae*	S	–	–	0	–	–	0	0	0	–	0	2	0	0	0	–	–	–	2 (11.1)
	R	–	–	2	–	–	2	2	2	–	2	0	2	2	2	–	–	–	16 (88.9)
NLF-GNB	S	–	–	1	–	–	1	1	–	–	1	1	0	0	–	1	–	–	6 (28.6)
	R	-	–	3	–	–	3	3	–	–	2	0	3	1	–	0	–	–	15 (71.4)
Total	S	9	4	3	2	3	2	6	1	3	6	28	7	18	7	8	19	12	138 (34.8)
	R	13	10	25	8	5	24	26	20	4	22	10	36	32	4	12	4	4	259 (65.2)

Abbreviations: *PEN* penicillin, *AMX* amoxicillin, *AUG* Augmentin, *PIP* piperacillin, *OXA* oxacillin, *CAZ* ceftazidime, *CRO* ceftriaxone, *CFZ* cefuroxime, *CXT* Cefoxitin, *CIP* ciprofloxacin, *MEM* Meropenem, *COT* co-trimoxazole, *GEN* gentamycin, *TOB* tobramycin, *AMK* amikacin, *CAF* chloramphenicol, *VAN* vancomycin, *NLF-GNB* non-lactose fermenter Gram-negative bacteria, *spp.* species, *S* sensitive, *R* resistant

### Hematological parameters among case and control groups

The mean of TLC among the case and control groups was 11.63 + 6.680 and 14.41 + 5.459, respectively. Similarly, the mean of ANC among cases and control groups was 7.34 + 5.706 and 9.47 + 3.570, respectively. Furthermore, the mean hemoglobin (Hgb) value among the case and control groups was 14.50 + 4.325 and 15.95 + 3.688, respectively. In this study, the mean of TLC among EOS study participants was 11.01 + 5.581 and 14.41 + 5.459, and in LOS it was 11.89 + 7.105 and 14.41 + 5.459, respectively. Also, the mean of ANC between the EOS case and the control group was 7.34 + 4.706 and 9.47 + 3.570, respectively. In LOS, the mean of ANC among the cases and control group was 7.34 + 6.103 and 9.47 + 3.570, respectively (Table 3).

**Table 3:** Hematological parameter characteristics among case and control study participants at UoG-CSH Northwest Ethiopia in 2021, *N* = 250

Variables	Case Mean + SD	Control Mean + SD	*P* value
Hematological parameter among neonatal sepsis study participants, *N* = 125			
WBC/mm^3^	11.63 + 6.68	14.41 + 5.46	0.000
RBC/mm^3^	5.73 + 15.84	4.73 + 0.92	0.483
Hemoglobin (g/dl)	14.50 + 4.33	15.95 + 3.69	0.005
Platelet count/mm^3^	209.84 + 158.94	227.2 + 138.17	0.358
Lymphocyte %	34.11 + 18.25	29.64 + 15.21	0.036
Neutrophil %	55.75 + 19.75	59.19 + 17.25	0.144
Lymphocyte/mm^3^	3.81 + 2.38	4.33 + 4.58	0.261
ANC/mm^3^	7.34 + 5.71	9.47 + 3.57	0.000
MCV/fl	97.70 + 32.67	98.13 + 9.48	0.890
Hematological parameter among EOS study participants *N* = 37			
WBC/mm^3^	11.01+ 5.581	14.41 + 5.46	0.001
RBC/mm^3^	4.65 + 1.203	4.73 + 0.92	0.647
Hemoglobin (g/dl)	16.12 + 4.480	15.95 + 3.69	0.814
Platelet count/mm^3^	166.62 + 127.547	227.20 + 138.17	0.018
Lymphocyte %	27.11 + 17.401	29.64 + 15.21	0.392
Neutrophil %	63.75 + 19.625	59.19 + 17.25	0.173
Lymphocyte/mm^3^	2.98 + 1.372	4.33 + 4.58	0.080
ANC/mm^3^	7.34 + 4.706	9.47 + 3.57	0.004
MCV/fl	94.75 + 16.098	98.13 + 9.48	0.112
Hematological parameter among LOS study participants *N* = 88			
WBC/mm^3^	11.89 + 7.11	14.41 + 5.46	0.004
RBC/mm^3^	6.18 + 18.88	4.73 + 0.92	0.391
Hemoglobin (g/dl)	13.81 + 4.09	15.95 + 3.69	0.000
Platelet count/mm^3^	228.01 + 167.77	227.20 + 138.17	0.969
Lymphocyte %	37.05 + 17.89	29.64 + 15.21	0.001
Neutrophil %	52.38 + 18.91	59.19 + 17.25	0.007
Lymphocyte/mm^3^	4.16 + 2.63	4.33 + 4.58	0.751
ANC/mm^3^	7.34 + 6.10	9.47 + 3.57	0.002
MCV/fl	98.95 + 37.53	98.13 + 9.48	0.815

### Sensitivity and specificity of hematological parameters

The sensitivity of TLC, Hgb, lymphocyte count, and ANC in the diagnosis of neonatal sepsis were 64.8, 68, 33.6, and 49.6%, respectively. The specificities of TLC, Hgb, lymphocyte count, and ANC for diagnosing neonatal sepsis were 64.8, 53.6, 83.2, and 90.4%, respectively. The sensitivity of TLC, Hgb, and ANC for diagnosing EOS were 73, 54.1, and 70.3%, respectively, and the specificities were 67.2, 70.4, and 58.4%, respectively. In addition, the sensitivity of TLC, Hgb, lymphocyte count, neutrophil percentage, and ANC for diagnosing LOS was 35.2, 43.2, 65.9, 31.8, and 51.1%, respectively. However, the specificity in LOS diagnosis was 92, 80, 50.4, 86.4, and 90.4%, respectively (Error! Reference source not found. and Table 4).

**Table 4:** Sensitivity and specificity of hematological parameters for the diagnosis of neonatal sepsis, EOS, and LOS

Screening parameters	Sensitivity (%)	Specificity (%)
**Comparison of hematological parameters for neonatal sepsis diagnosis,** ***N*** **= 125**		
WBC/mm^3^ (< 12.5)	64.8	64.8
Hemoglobin (g/dl) (< 16.86)	68	53.6
Lymphocyte % (> 41.4)	33.6	83.2
ANC/mm^3^ (< 5.5)	49.6	90.4
**Comparison of hematological parameters for EOS diagnosis*, N = 37***		
WBC/mm^3^ (< 11.65)	73	67.2
Platelet count (145)	54.1	70.4
ANC/mm^3^ (8.45)	70.3	89.6
**Comparison of hematological parameters for LOS diagnosis,** ***N*** **= 88**		
WBC/mm^3^ (< 9.05)	35.2	92
Hemoglobin (g/dl) (< 13.2)	43.2	80
Lymphocyte % (> 28.85)	65.9	50.4
Neutrophil % (41.5%)	31.8	86.4
ANC/mm^3^ (< 5.55)	51.1	90.4

## Discussion

Neonatal sepsis is a life-threatening disorder that is responsible for about 15% of all neonatal deaths in developing countries [22, 23]. It is characterized by signs and symptoms of microbial infection, usually bacteria, in the first month of life [24]. However, accurate diagnosis is difficult for clinicians and may lead to inappropriate diagnosis [4]. Which may increase the risk of drug resistance and its adverse effects [24, 25]. Therefore, this study aimed to assess the role of CBC parameters in diagnosing neonatal sepsis.

In this study, the leading causative agents of neonatal sepsis were CoNS*, K. pneumonia, S. aureus, S. viridians,* and *E. coli,* with 19.6, 10.4, 4.0, 3.2, and 2.8%, respectively. These findings demonstrate that GPB accounted for 58.4% of the causative agents, while GNB accounted for 41.6%. The current study was in line with studies conducted in India [22] and Nigeria [26] which showed GPB as the predominant isolate. In contrast, a study conducted in India showed the predominant infection was due to GNB [2].

In this study, the mean of TLC among case groups (11.63 + 6.68) was lower in sepsis patients as compared to the control group (14.41 + 5.459). Besides, the mean of ANC is lower in the case group (7.34 + 5.71) than in the control groups (9.47 + 3.57). This finding showed that neonates with sepsis suffer from leucopenia and neutropenia. In microbial infections, neutrophils can quickly be depleted, inhibiting the bone marrow from producing more neutrophils and promoting neutrophil adhesion to the altered endothelium cells [27, 28]. Furthermore, the mean platelet count in the case group was lower (209.84 + 158.94) than in the control group (227.2 + 138.17). Thrombocytopenia in response to products of microorganisms causing neonatal sepsis could be the reason for the lower platelet count. Microbial products can cause platelet clumping and adherence, leading to platelet destruction [29]. Similar to the current study, a report in Pakistan found that leucopenia, neutropenia, and thrombocytopenia were more often found in the neonatal sepsis cases than in the control group [30].

In the current study, leucopenia in diagnosing sepsis among neonates (less than or equal to 12,500/mm^3^) has a sensitivity and specificity of 64.8%. Besides, the sensitivity and specificity of leucopenia in diagnosing EOS were 73 and 67.2%, respectively. Similarly, leucopenia among study participants with LOS has a sensitivity of 35.2% and a specificity of 92%. These findings showed that leucopenia has a better performance on EOS detection than LOS. Similarly, the specificity of leucopenia in the diagnosis of neonatal sepsis can be considered a good diagnostic marker. This is due to neonates with less than 3 days of age having no response mechanism to replace the consumed leucocytes. The current study finding can be considered higher than a study conducted in Nigeria that showed the sensitivity of leucopenia was 46%. In contrast, this study has lower specificity and sensitivity than studies conducted in Bangladesh [31], India [29], and Egypt [25] where the sensitivity was 97.56, 80.76, and 90.0%, respectively. Besides, the specificity in Bangladesh [31], India [29], and Egypt [25] was 72.97, 90.0, and 93.3%, respectively. The variation may be due to differences in sample size, method of WBC measurement, blood sampling time, and the severity of infection [2].

In the current study, the sensitivity and specificity of neutropenia (< 5500) were 49.6 and 90.4%, respectively. Besides, the ANC sensitivity in the diagnosis of EOS was 70.3% and the specificity was 58.4%. The diagnostic capacity of ANC on LOS was 51.1% sensitivity and 90.4% specificity. When we compared this study with a study conducted in Nigeria, it showed that the ANC had a sensitivity of 96% and a specificity of 25% [32]. In India, the ANC sensitivity was 84.61% and the specificity was 79.72% [29]. In contrast, a study conducted in India showed low sensitivity of ANC [33, 34]. In Egypt, the ANC sensitivity was 97.5% and the specificity was 36.7% [25]. The neutrophil count can be considered a good marker, but if it is used alone, it may be misleading because it can be affected by PIH, asphyxia neonatorum, isoimmune neutropenia, congenital neutropenia, and certain inborn errors of metabolism [35, 36]. Neutropenia has been more common in association with sepsis compared with neutrophilia, probably because of increased adherence to altered endothelial cells and utilization at the site of infection. The elevation of PMN is often very late and inconsistent [24].

In the current study, the sensitivity and specificity of thrombocytopenia (< 145,000) were 54.1 and 70.4%, respectively. In neonates with sepsis, thrombocytopenia is common, and usually associated with disseminated intravascular coagulation, increased platelet destruction, sequestration secondary to infections, failure in platelet production due to reduced megakaryocytes [37], and damaging effects of endotoxin [38, 39]. So, reduction of the platelet count can be used for the diagnosis of neonatal sepsis. The current finding is higher than a study conducted in Bangladesh, which was 27.31%. In contrast, the sensitivity of thrombocytopenia in the current study was lower than in reports of Nigeria [32], India [29], Egypt [25], and India [2], which was 90, 73.07, 100.0, and 73.68%, respectively. The specificity of thrombocytopenia was higher than a study conducted in Nigeria [32], India [29], Egypt [25], and India [2], which were 56, 66.21, 56.7, and 53.09%, respectively. In contrast, a Bangladesh study found that the specificity was 89.63% [31]. Therefore, thrombocytopenia in early neonates can be taken as a good predictor of neonatal septicemia [34].

### Strength and limitations

The strength of this study is that it determines the complete blood cell count and blood culture for both cases and controls. However, the study has its limitations. The study used a cross-sectional study design, which has a chicken-egg dilemma. Second, we used a convenient sampling technique, which may have affected the result.

## Conclusion

Hematological parameters such as TLC, ANC, and platelet count are significantly lower in the case than in the control. Also, hematological parameters like TLC, ANC, and platelet count can be considered as important tools for the diagnosis of neonatal sepsis. Therefore, these parameters can be used as a diagnostic tool in resource-limited areas in the diagnosis of neonatal sepsis.

## Data Availability

All data generated and/or analyzed in this study are available within the manuscript.

## Abbreviations

ANC: Absolute neutrophil count
CBC: Complete blood count
CoNS: Coagulase-negative Staphylococcus
EOS: Early-onset sepsis
GNB: Gram-negative bacteria
GPB: Gram-positive bacteria
Hgb: Hemoglobin
IQR: Interquartile Range
LOS: Late-onset sepsis
ROC: Receiver operating characteristic
SPSS: Statistical Package for Social Science
TAT: Turnaround time
TLC: Total leucocyte count
UoG-CSH: University of Gondar Comprehensive Specialized Hospital.
